# Human Cases of *Borrelia miyamotoi* Disease, Slovenia, 2025

**DOI:** 10.3201/eid3208.260326

**Published:** 2026-08

**Authors:** Petra Bogovič, Jan Slunečko, Meta Kodre, Rok Kogoj, Mateja Brecl Jakob, Miša Korva, Eva Ružić-Sabljić, Franc Strle

**Affiliations:** University Medical Center Ljubljana, Ljubljana, Slovenia (P. Bogovič, F. Strle); University of Ljubljana, Ljubljana (J. Slunečko, M. Kodre, R. Kogoj, M. Brecl Jakob, M. Korva, E. Ružić-Sabljić)

**Keywords:** Borrelia miyamotoi, bacteria, ticks, vector-borne infections, febrile illness, Slovenia, *Suggested citation for this article*: Bogovič P, Slunečko J, Kodre M, Kogoj R, Brecl Jakob M, Korva M, et al. Human cases of *Borrelia miyamotoi* disease, Slovenia, 2025. Emerg Infect Dis. 2026 Aug [*date cited*]. https://doi.org/10.3201/eid3208.260326

## Abstract

We identified human *Borrelia miyamotoi* infections in Slovenia in 2 of 337 adults with undifferentiated fever tested positive by metagenomic sequencing and PCR. Both patients reported recent local tick bites. The illness was mild and self-limited. Our findings underscore the need to consider this pathogen in evaluating fever after tick bite.

*Borrelia miyamotoi* is an emerging tickborne spirochete belonging to the relapsing fever group of *Borrelia* spp. It was first detected in *Ixodes persulcatus* ticks in Japan and formally described as a novel species in 1995 (*1*). The first human cases of *B. miyamotoi* disease were reported in Russia in 2011, establishing its clinical relevance as a human pathogen (*2*). Since then, *B. miyamotoi* has been identified in *Ixodes* ticks and documented in human cases throughout the Northern Hemisphere, including Asia, North America, and Europe (*3*).

In Europe, the prevalence of *B. miyamotoi* in *I. ricinus* ticks varies geographically; estimated prevalence is 1%–1.5% (*3*,*4*), and the reported prevalence up to 4% (*5*). In Slovenia, *B. miyamotoi* was detected in 8 (2%) of 398 tested *I. ricinus* ticks (*6*) and in 2% of tested small rodents (*7*). Seroprevalence studies have demonstrated *B. miyamotoi* antibodies in an average of 4.4% of tested persons; reported values were 0%–25.6% (*3*). However, the lack of a commercially available serologic assay limits comparability across studies. A previous study examined exposure to and infection with *B. miyamotoi* in the Netherlands and Sweden during 2007–2019 (*8*). A total of 2,160 participants, including healthy controls, persons with a recent tick bite, and patients with post–tick bite fever, were tested by multiantigen serologic assay. IgM or IgG seroprevalence was 1.0%–2.5% in healthy participants, 6.1%–8.9% in those reporting recent tick bites, and as high as 16.5% in febrile persons in Sweden. 

By March 1, 2021, more than 500 cases of *B. miyamotoi* disease had been reported worldwide, primarily in Asia (particularly Russia) and North America, whereas 6 cases had been documented in Europe (*3*). A subsequent literature search through January 31, 2026, identified 3 additional cases in Europe (*9*–*11*); those cases originated from the Netherlands, Germany, Sweden, Austria, Norway, and Poland (*3*,*9*–*11*).

*B. miyamotoi* disease usually manifests as acute febrile illness with fatigue, headache, chills, myalgia, arthralgia, and nausea. Although the pathogen belongs to the relapsing fever group, recurrent febrile episodes occur in ≈10% of patients (*3*). Meningoencephalitis has been reported predominantly in immunocompromised patients (*3*,*9*,*10*,*12*). Here, we describe 2 cases of *B. miyamotoi* disease acquired and diagnosed in Slovenia.

## The Study

Participants qualified for our study if they met the following criteria: adult patients with acute febrile illness (>38°C for >2 days), without localizing signs, who remained without diagnosis after standard laboratory testing and were evaluated at the Department of Infectious Diseases, University Medical Centre Ljubljana (Ljubljana, Slovenia), during 2021–2025. We analyzed archived EDTA blood samples from those patients using shotgun metagenomic sequencing (mNGS) as previously described (*13*). The National Medical Ethics Committee of the Republic of Slovenia approved the use of archived specimens (approval no. 0120-253/2023/3).

We detected *B. miyamotoi* in 2 (0.6%) of 337 cases by mNGS. We confirmed the presence of *B. miyamotoi* in both samples by specific real-time PCR (*2*) and sequencing of the full-length 16S rRNA gene (Figure). In addition to the 2 cases of *B. miyamotoi*, we identified other pathogens among sequenced patients: parvovirus B19 (n = 3), Epstein-Barr virus (n = 1), tick-borne encephalitis virus (n = 1), *Anaplasma phagocytophilum* (n = 1), *Neoehrlichia mikurensis* (n = 1), and *Spiroplasma ixodetis* (n = 15).

**Figure:**
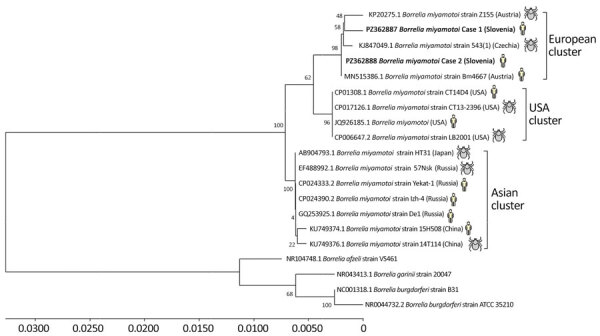
Phylogenetic analysis of 2 cases of *Borrelia miyamotoi* disease, Slovenia, 2025. Analysis was based on the 16S rRNA gene. We aligned consensus sequences (1,241-bp-long 16S rRNA sequences) using MAFFT version 7.490 (https://mafft.cbrc.jp/alignment/software) with a global alignment >1,000 iterations. We constructed the phylogenetic tree based on the multiple sequence alignment using IQ-TREE 2 version 2.2.0 (http://www.iqtree.org) with automatic model selection for tree inference and ultrafast bootstrapping with 1,000 iterations. Bold text indicates study sequences (GenBank accession nos. PZ362887 and PZ362888). We selected reference sequences to represent the European, Asian, and US *B. miyamotoi* clusters. We used *B. burgdorferi* (GenBank accession nos. NC_001318.1, NR_044732.2), *B. afzelii* (GenBank accession no. NR_104748.1) and *B. garinii* (GenBank accession no. NR_043413.1) as the outgroups. Numbers at nodes indicate bootstrap values. Scale bar indicates substitutions per site.

Case 1 was in a 36-year-old man who sought care in July 2022 for a 3-day history of fever as high as 38 °C and moderate headache, accompanied by occasional dry cough and mild nausea. Three weeks earlier, he had removed 2 ticks and noticed no subsequent skin changes. His medical history was unremarkable, and he was not taking any regular medications. He had not traveled outside Slovenia in the preceding 6 months. In 2012, he had received the basic vaccination for tick-borne encephalitis.

At examination, he was afebrile (36°C); blood pressure was 111/73 mm Hg, pulse 50 bpm, and peripheral oxygen saturation 98%. Physical examination was unremarkable; the patient had no rash or meningeal signs. Laboratory testing revealed several abnormalities, including elevated inflammatory markers (C-reactive protein 61 mg/L [reference range <5 mg/L]; procalcitonin 1.93 µg/L [reference range <0.25 µg/L]), increased creatinine (127 µmol/L [reference range 49–90 µmol/L]), mild thrombocytopenia (136 × 10^9^/L [reference range 150–410 × 10^9^/L]), abnormal liver function tests (total bilirubin 68 µmol/L [reference range 3–22 µmol/L]; aspartate aminotransferase 0.99 µkat/L [reference range <0.52 µkat/L]; alanine aminotransferase 1.24 µkat/L [reference range <0.57 µkat/L]; γ-glutamyl transferase 1.24 µkat/L [reference range <0.63 µkat/L]), and increased lactate dehydrogenase (5.93 µkat/L [reference range <4.12 µkat/L]). Chest radiography findings were unremarkable. Molecular and serologic tests for *A. phagocytophilum*, hantaviruses (the patient sought care during an ongoing hantavirus outbreak in Slovenia), and SARS-CoV-2 were negative. IgM and IgG serology for *Borrelia burgdorferi* sensu lato were also negative. He received no antimicrobial therapy. At 2-month follow-up, he was clinically well, and laboratory abnormalities had resolved.

Case 2 was in a 31-year-old previously healthy woman who, in May 2025, experienced acute fever (38.5 °C), chills, nausea, severe frontotemporal headache (visual analogue scale 8/10), photophobia, neck and shoulder pain, and lower limb paresthesias. She had removed a tick 10 days earlier. She had not traveled outside Slovenia in the preceding 6 months.

At examination, she was alert and afebrile (35.9°C) and had unremarkable vital signs (blood pressure 101/62 mm Hg, heart rate 88 bpm, peripheral oxygen saturation 100%). Mild neck pain on anteflexion and horizontal nystagmus were observed; the remainder of the neurologic and physical examination was unremarkable. Laboratory testing showed elevated C-reactive protein (37 mg/L), leukopenia (leukocytes 3.2 × 10^9^ cells/L [reference range 4–10 × 10^9^ cells/L]) with lymphopenia (lymphocytes 0.46 × 10^9^ cells/L [reference range 1.1–3.5 × 10^9^ cells/L]), and mild anemia (hemoglobin 116 g/L [reference range 120–150 g/L]). Renal function test results, electrolytes, and coagulation parameters were within reference ranges. Molecular and serologic analyses for *A. phagocytophilum*, along with serologic testing for tick-borne encephalitis virus, and *B. burgdorferi* s.l. yielded negative results. She received treatment for symptoms only. At 1-month follow-up, she was asymptomatic, and her laboratory values were within reference ranges.

## Conclusions

We report 2 confirmed human cases of *B. miyamotoi* disease in Slovenia, both locally acquired. Those cases add to the small but growing number reported in Europe and provide clinical confirmation of human infection in a region where *B. miyamotoi* has been documented in ticks. Both patients were immunocompetent adults who experienced a self-limiting febrile illness without neurologic complications and without relapses; neither was treated with antimicrobial drugs. Detection by mNGS, confirmed by *B. miyamotoi*–specific PCR, underscores the importance of molecular diagnostics for identifying emerging tickborne pathogens in patients with unexplained febrile illness.

Information on asymptomatic or self-limiting *B. miyamotoi* infections remains scarce. The true incidence of *B. miyamotoi* disease is unknown because of limited clinical awareness, nonspecific and often mild symptoms, and restricted availability of diagnostic tools (*3*). In Slovenia, where tick-borne encephalitis and human granulocytic anaplasmosis represent the most common febrile illnesses after tick bites (*14*,*15*), clinicians should consider *B. miyamotoi* disease in the differential diagnosis. Our findings expand knowledge of the pathogen’s geographic distribution and highlight the need for enhanced surveillance and improved diagnostics to better define disease burden in Slovenia and neighboring countries.

## References

[1] Fukunaga M, Takahashi Y, Tsuruta Y, Matsushita O, Ralph D, McClelland M, et al. Genetic and phenotypic analysis of *Borrelia miyamotoi* sp. nov., isolated from the ixodid tick *Ixodes persulcatus*, the vector for Lyme disease in Japan. Int J Syst Bacteriol. 1995;45:804–10. 10.1099/00207713-45-4-804 7547303

[2] Platonov AE, Karan LS, Kolyasnikova NM, Makhneva NA, Toporkova MG, Maleev VV, et al. Humans infected with relapsing fever spirochete *Borrelia miyamotoi*, Russia. Emerg Infect Dis. 2011;17:1816–23. 10.3201/eid1710.101474 22000350 PMC3310649

[3] Hoornstra D, Azagi T, van Eck JA, Wagemakers A, Koetsveld J, Spijker R, et al. Prevalence and clinical manifestation of *Borrelia miyamotoi* in *Ixodes* ticks and humans in the northern hemisphere: a systematic review and meta-analysis. Lancet Microbe. 2022;3:e772–86. 10.1016/S2666-5247(22)00157-4 36113496

[4] Hansford KM, Wheeler BW, Tschirren B, Medlock JM. Questing *Ixodes ricinus* ticks and *Borrelia* spp. in urban green space across Europe: a review. Zoonoses Public Health. 2022;69:153–66. 10.1111/zph.12913 35122422 PMC9487987

[5] Snegiriovaitė J, Lipatova I, Razgūnaitė M, Paulauskas A, Radzijevskaja J. Prevalence and diversity of *Borrelia* spp. in questing ticks from urban green spaces in Lithuania. Ticks Tick Borne Dis. 2025;16:102512. 10.1016/j.ttbdis.2025.102512 40580570

[6] Šušnjar J, Cerar Kisek T, Strasek Smrdel K, Ruzic-Sabljic E, Adam K, Ivovic V. Detection, identification and genotyping of *Borrelia* spp. in ticks of Coastal-Karst and Littoral-Inner Carniola regions in Slovenia. Folia Parasitol. 2023;70:2023.007. 10.14411/fp.2023.007 37042198

[7] Cerar T, Korva M, Avšič-Županc T, Ružić-Sabljić E. Detection, identification and genotyping of *Borrellia* spp. in rodents in Slovenia by PCR and culture. BMC Vet Res. 2015;11:188. 10.1186/s12917-015-0501-y 26253121 PMC4529734

[8] Hoornstra D, Stukolova OA, van Eck JA, Sokolova MI, Platonov AE, Hofhuis A, et al. Exposure, infection and disease with the tick-borne pathogen *Borrelia miyamotoi* in the Netherlands and Sweden, 2007–2019. J Infect. 2024;89:106326. 10.1016/j.jinf.2024.106326 39454832

[9] Schwartz T, Hoornstra D, Øie E, Hovius J, Quarsten H. Case report: first case of *Borrelia miyamotoi* meningitis in an immunocompromised patient in Norway. IDCases. 2023;33:e01867. 10.1016/j.idcr.2023.e01867 37577049 PMC10412827

[10] Dambietz CA, Kintzinger T, Schuler F, Albers A, Suntrup-Krueger S, Fingerle V, et al. Nanopore sequencing identifies *Borrelia miyamotoi* as an unexpected cause of meningitis after B cell depletion. Neuropathol Appl Neurobiol. 2024;50:e13017. 10.1111/nan.13017 39511958 PMC11618485

[11] Fiecek B, Szewczyk T, Lewandowska G, Chmielewski T. *Borrelia miyamotoi* DNA in a patient suspected of Lyme borreliosis. Ann Agric Environ Med. 2025;32:142–5. 10.26444/aaem/191046 40159748

[12] Kubiak JM, Klevay M, Hilt EE, Ferrieri P. Acute meningoencephalitis associated with *Borrelia miyamotoi*, Minnesota, USA. Emerg Infect Dis. 2024;30:1472–4. 10.3201/eid3007.231611 38916722 PMC11210636

[13] Slunečko J, Kogoj R, Zakotnik S, Suljič A, Knap N, Bosilj M, et al. Development and performance evaluation of a clinical metagenomics approach for identifying pathogens in the whole blood from patients with undifferentiated fever. Front Cell Infect Microbiol. 2025;15:1667422. 10.3389/fcimb.2025.1667422 41031110 PMC12477237

[14] National Institute for Public Health. Monitoring infectious diseases transmitted by arthropods in Slovenia in 2024 [cited 2026 Feb 12]. https://nijz.si/publikacije/spremljanje-nalezljivih-bolezni-ki-jih-prenasajo-clenonozci-v-sloveniji-v-letu-2024

[15] Lotrič-Furlan S, Rojko T, Jelovšek M, Petrovec M, Avšič-Županc T, Lusa L, et al. Comparison of clinical and laboratory characteristics of patients fulfilling criteria for proven and probable human granulocytic anaplasmosis. Microbes Infect. 2015;17:829–33. 10.1016/j.micinf.2015.09.017 26432519

